# Underreplicated Regions in *Drosophila melanogaster* Are Enriched with Fast-Evolving Genes and Highly Conserved Noncoding Sequences

**DOI:** 10.1093/gbe/evu156

**Published:** 2014-07-24

**Authors:** Igor V. Makunin, Tatyana D. Kolesnikova, Natalya G. Andreyenkova

**Affiliations:** ^1^Research Computing Centre, The University of Queensland, St Lucia, Queensland, Australia; ^2^Institute of Molecular and Cellular Biology of the Siberian Branch of the Russian Academy of Sciences, Novosibirsk, Russia; ^3^Novosibirsk State University, Russia

**Keywords:** *Drosophila melanogaster*, replication timing, fast-evolving genes

## Abstract

Many late replicating regions are underreplicated in polytene chromosomes of *Drosophila melanogaster*. These regions contain silenced chromatin and overlap long syntenic blocks of conserved gene order in drosophilids. In this report we show that in *D. melanogaster* the underreplicated regions are enriched with fast-evolving genes lacking homologs in distant species such as mosquito or human, indicating that the phylogenetic conservation of genes correlates with replication timing and chromatin status. Drosophila genes without human homologs located in the underreplicated regions have higher nonsynonymous substitution rate and tend to encode shorter proteins when compared with those in the adjacent regions. At the same time, the underreplicated regions are enriched with ultraconserved elements and highly conserved noncoding sequences, especially in introns of very long genes indicating the presence of an extensive regulatory network that may be responsible for the conservation of gene order in these regions. The regions have a modest preference for long noncoding RNAs but are depleted for small nucleolar RNAs, microRNAs, and transfer RNAs. Our results demonstrate that the underreplicated regions have a specific genic composition and distinct pattern of evolution.

## Introduction

Metazoan genomes can be divided into long regions of similar properties. At least half of *Drosophila melanogaster* genes are organized in long domains spanning up to several dozens genes (de Wit et al. 2008). Domains contain different types of chromatin and may be defined by gene density, transcription, and insulator proteins (Hou et al. 2012). Domains can be characterized by specific proteins such as LAM (Shevelyov et al. 2009), groups of proteins (Filion et al. 2010; Kharchenko et al. 2011), or replication timing (Schwaiger et al. 2009). These features are interdependant, for example, early and late replication domains correlate with chromatin architecture (Eaton et al. 2011) or gene density (Hiratani and Gilbert 2009; Belyakin et al. 2010). Although localization of early and late replication domains is not identical in different cell types (Hiratani et al. 2008; Schwaiger et al. 2009), the replication timing is remarkably conserved between distant species such as human and mouse (Yaffe et al. 2010). In human and mouse, the replication timing depends on Rif1 (Cornacchia et al. 2012; Yamazaki et al. 2012). In flies, the SUUR protein regulates the late replication in polytene chromosomes and endoreplication (Volkova et al. 2003; Zhimulev et al. 2003).

Late and early replication domains evolve in somewhat different patterns. The divergence at synonymous sites and putatively unconstrained intronic sites is elevated in the late replication sequences in the *D. melanogaster* genome (Weber et al. 2012). Late replicating regions associate with the duplication hotspots, whereas deletions prevail in early replicating regions (Cardoso-Moreira and Long 2010; Cardoso-Moreira et al. 2011).

In drosophilids, gene order is conserved in many late replicating regions (Andreyenkova et al. 2013). Regions with the conserved gene order tend to bind LAM and SUUR (Ranz et al. 2012). LAM plays an important role in chromosome and nucleus structure (Shevelyov and Nurminsky 2012). Preservation of gene order in some genomic regions may also be attributed to a complex regulatory network of distant elements, such as enhancers (Kikuta et al. 2007; Hufton et al. 2009). The association between highly conserved elements and their potential target genes is maintained in distant vertebrates (Sun et al. 2008). Highly conserved noncoding elements (HCNEs) are overrepresented in long syntenic blocks (Engstrom et al. 2007), and the 21 longest homologous collinear blocks in the *D. melanogaster* genome are enriched with HCNEs (von Grotthuss et al. 2010). However, the disruption of a large syntenic block did not produce any severe phenotype (Diaz-Castillo et al. 2012), and no evidence was found for selection maintaining clusters of coexpressed genes in drosophilids (Weber and Hurst 2011).

Many very late replicating regions are underreplicated in polytene tissues (Zhimulev et al. 2003). Mutation of *SuUR* gene abolishes the underreplication, whereas additional copies of the gene increase the number of UnderReplicated regions (URs) (Zhimulev et al. 2003). Using cDNA microarrays, Belyakin et al. (2005) identified 52 URs in the fly strain with four copies of *SuUR* gene. Additional URs were identified in salivary gland, fat body, and gut using high-density olygonucleotides microarrays (Nordman et al. 2011; Sher et al. 2012). URs tend to overlap with silenced chromatin. Of the five major types of drosophila chromatin (Filion et al. 2010), URs show significant overlap with BLACK (silenced) chromatin but contain essentially no active chromatin (Belyaeva et al. 2012). The proportion of transgenes with a partially silenced marker gene is higher in URs than in flanking regions (Babenko et al. 2010).

URs were identified using a sliding window of ten genes (Belyakin et al. 2005), so the URs’ borders are defined by the position of genes, and the precision of mapping depends on the window size. Belyaeva et al. (2012) combined chromatin signatures with URs and established borders for 60 regions. We refer to these 60 regions as UR(B). A subsequent study (Andreyenkova et al. 2013) demonstrated that many UR(B) regions overlap long syntenic blocks with conserved gene order (von Grotthuss et al. 2010). In fact, in many cases, the syntenic blocks are nearly identical to the corresponding UR(B) regions.

In this work, we analyzed DNA conservation in 60 UR(B) regions and found that these regions contain a very high proportion of genes without homologs in distant species such as mosquito or human but are enriched in highly conserved noncoding sequences, especially in the introns of some long genes. Our results indicate that the phylogenetic conservation of genes correlates with replication timing.

## Materials and Methods

The coordinates of the UR(B) (Belyaeva et al. 2012) for BDGP Release 5/dm3 genome assembly are listed in supplementary data set S1, Supplementary Material online. The FlyBase Genes (version 5.12) were assigned to UR(B) through the UCSC Table Browser (Karolchik et al. 2004) if at least 50% of its genomic length between the left-most Start and right-most End of all transcript isoforms was covered by UR(B). The protein-coding FlyBase Genes 5.12 annotation for the dm3 genome assembly was downloaded from the UCSC Genome Browser web site (Meyer et al. 2013). The proteins (single isoform per locus, first according to alphabetical order) were downloaded from linked table dm3.flyBasePep and the length was calculated by LEN function in Excel.

The drosophila genes with homologs in distant species were selected using the UCSC Table Browser (Karolchik et al. 2004). The FlyBase Genes 5.12 with human homologs were selected from hgBlastTab table (identity, length of alignment, eValue) using “Selected fields from primary and related tables” output format. In several cases, multiple isoforms of one gene aligned to the human genome so only one transcript with the lowest eValue for alignment was kept. The *D. melanogaster* genes with homologs in mosquito *Anopheles gambiae* (anoGam1) and *D. virilis* (droVir3) were identified by presence of CDS FASTA alignments in multiz15way (dm3 centric) MAF table. Gene Ontology was analyzed using GOrilla (Eden et al. 2009), with two unranked lists of genes. The UR(B) genes with homologs in distant species were analyzed against all genes conserved in the same species, and UR(B) genes without homologs were analyzed against all genes without homologs in the same species.

The alignment of genes in UR(B) and adjacent regions were extracted from MAF blocks 15-way multiz (dm3) on Galaxy (Giardine et al. 2005) as follows: 

A BED file of FlyBase Genes 5.12 (12 fields) with a single transcript isoform per locus (first according to alphabetical order) was exported from the UCSC Table Browser to Galaxy. The dm3-centric alignments of coding sequences for each gene were extracted by “Stitch Gene blocks” (version 1.0.1) from the “Fetch Alignments” menu without splitting into gapless MAF blocks for the following assemblies of species from Sophophora subgenus: dm3, droSim1, droYak2, droEre2, droAna3, dp4, droPer1, and droWil1. The alignments were joined together by “Concatenate FASTA alignment by species” from the “FASTA manipulation” menu, and the alignment width was changed to 60-bp blocks by “FASTA Width formatter” from the “FASTA manipulation” menu. The resulted FASTA files were imported in MEGA4 and pairwise distances were calculated using the Pamilo–Bianchi–Li method (Pamilo and Bianchi 1993; Tamura et al. 2007).

For FlyBase noncoding data set, we downloaded selected fields from the primary table dm3.flyBaseNoncoding and symbolic gene names (symbol) from linked table dm3.flyBase2004Xref using UCSC Table Browser. The symbolic names were used for selection of transfer RNAs (tRNAs) and small nucleolar RNAs (snoRNAs).

The data for replication time in embryonic Kc cells and Cl8 cells derived from wing disks (Schwaiger et al. 2009) were downloaded from the Replication Domain site (http://www.replicationdomain.com/, last accessed August 1, 2014). The data for chromosomes X, 2, 3, and 4 were selected and a BED file was created with four columns (chr, start, end, and replication score) using “awk.” The BED file was uploaded to Galaxy and intersected with loci (based on FlyBase Genes 5.12 annotation) using the “Join” command from “Operate on Genomic Intervals” menu with requirement of at least 5-bp overlap. The average replication time for all probes overlapping every locus was calculated by “Group” command in “Join, Subtract and Group” menu.

For syntenic conservation, we used Gene Order (GO) model (von Grotthuss et al. 2010). Testis-specific genes were extracted from the FlyAtlas data set (http://www.flyatlas.org/, last accessed August 1, 2014) and provided to us by Stepan Belyakin. The BNA precursors (release 19, August 2012) were downloaded from miRBase (Kozomara and Griffiths-Jones 2011).

The expected number of the Sophophora ultraconserved elements (UCEs) (Makunin et al. 2013) in UR(B) was calculated proportional to the length of the corresponding annotation (exonic, intronic, intergenic, FlyBase Genes 5.12). The UCEs were defined as sequences at least 100 bp long, and hence cannot be mapped into regions smaller than 100 bp. According to our test, it is appropriate to use all intronic or intergenic regions in UR(B) and outside regions for estimation of expected values because the proportion of such regions over 100 bp long in UR(B) and outside regions is essentially identical to that of all intronic and intergenic regions.

The phastCons elements (Siepel et al. 2005) were selected from the phastConsElements15way table. The HCNEs identified in pairwise comparison between *D. melanogaster* and *D. ananassae*, *D. pseudoobscura*, *D. mojavensis* or *D. virilis* (Engstrom et al. 2007) were downloaded from http://ancora.genereg.net/ (last accessed August 1, 2014). Common regions in all four data sets were selected using Base-pair-wise intersection (option AND) on the UCSC Table Browser. The elements shorter than 50 bp were filtered out, as well as elements overlapping exons of FlyBase genes 5.12. The final set contains 7,574 HCNEs.

Chi-squared test was calculated in Excel. For a 2 × 2 contingency table, we used online calculator (http://faculty.vassar.edu/lowry/tab2x2.html, last accessed August 1, 2014). The *t*-, *F*- and *U*-tests were calculated in R package (R Development Core Team 2012).

## Results

### Proportion of Genes with Homologs in Distant Species Is Smaller in UR(B)

UR(B) regions cover 14.8 Mb (12%) of the *D. melanogaster* genome on chromosomes X, 2, and 3 and contain 993 genes (supplementary data set S2, Supplementary Material online). We analyzed conservation of UR(B) genes in distant species, human and mosquito, using available information from the UCSC Genome Browser (see Materials and Methods for details). The proportion of the UR(B) genes with homologs in distant species is significantly smaller than the genomic average (table 1).

**Table 1:** The UR(B) Genes with Homologs in the Human and Mosquito Genomes

	All	Testis-Specific	Other
Genes	933	298	544
With homologs in human	104	15	80
Expected	400.2	27.9	270.0
Exp./Obs.	3.8	1.9	3.4
Chi-squared test *P* value	1.7E-85	0.01	1.1E-59
With homologs in mosquito	506	129	343
Expected	719.9	143.0	451.6
Exp./Obs.	1.4	1.1	1.3
Chi-squared test *P* value	2.1E-62	0.1	2.4E-35

The URs are enriched with testis-specific genes (Belyakin et al. 2005), and sex-related genes tend to evolve fast in drosophilids (Haerty et al. 2007). Only 9% of all testis-specific genes have known homologs in the human genome compared with 50% of all other genes with known expression pattern. We estimated the conservation status separately for testis-specific and “other” genes. Out of 842 UR(B) genes with known expression pattern, 298 (35%) are classified as testis-specific, a 2.7-fold increase over the proportion of such genes in the genome (Chi-squared test, *P* = 1.5E-84). Proportion of both testis-specific and “other” genes with human or mosquito homologs is smaller in UR(B) than the genomic average (table 1).

We checked whether it is possible to explain the overrepresentation of the fast-evolving genes in UR(B) by an “overoptimistic” or incorrect gene annotation in *D. melanogaster,* for example, withdrawn gene models or lack of evidence for transcription. We selected 64 FlyBase Genes in UR(B) without homologs in the *D. virilis* genome and queried FlyBase (McQuilton et al. 2012). All 64 genes were listed as current gene models (checked on February 4, 2013, FB2013_01 release r5.49), with one gene, *CG9284*, annotated as a noncoding RNA, *CR9284*. Next, we checked how many genes have expressed sequence tag (EST)-based evidence of expression in *D. melanogaster*. Out of 64 *D. melanogaster* genes without homologs in *D. virilis,* 53 (83%) overlap available ESTs in *D. melanogaster* compared with 90% (786 out of 869) for genes with homologs but the difference is on the border of statistical significance (2 × 2 contingency table, Pearson chi-square 3.84, *P* = 0.05). The majority of genes without homologs in distant drosophilids have evidence for transcription and are annotated as genes in current release of FlyBase, and apparently represent genuine gene models.

The Gene Ontology analysis revealed a significant enrichment for several categories for the nonconserved UR(B) genes (supplementary data set S1, Supplementary Material online). However, in many cases the enrichment is observed for gene clusters, such as *Osiris* and *Stellate* genes. The UR(B) genes conserved in distant species demonstrate enrichment for GO categories linked to membrane. The GO categories overrepresented in UR(B) generally contain just a few genes in UR(B) regions, for example, GO:0007424 (open tracheal system development), with the most significant noncorrected *P* value of 1.1E-05 for the genes with human homologs, contains just nine UR(B) genes, out of 96 with the assigned GO categories.

Our results indicate that the smaller proportion of genes in UR(B) with homologs in distant species correlates with a significant enrichment of these regions with the fast-evolving testis-specific genes as well as a low conservation of the “other” genes. This observation is somewhat surprising considering the conservation of gene order of UR(B) regions in drosophilids (Andreyenkova et al. 2013). This apparent contradiction can be explained by the approach used to define syntenic blocks, or orthologous landmarks (von Grotthuss et al. 2010). The authors excluded genes not annotated in subgenus *Drosophila* (*D. virilis*, *D. mojavensis, D. grimshawi*), genes located outside of syntenic blocks and not annotated in the studied species, and genes involved in complex rearrangements (von Grotthuss et al. 2010). As a result, the fast-evolving genes without homologs in distant species were excluded from the analysis. Although on main chromosomes 21.3% of genes were excluded, in UR(B) the proportion of excluded genes was 28.5%. Genes not annotated in distant drosophilids (*D. virilis*, *D. mojavensis, D. grimshawi*) comprise 71.5% of excluded genes on main chromosomes, whereas in UR(B) this category corresponds to 94.6% of the excluded genes. So, the majority of genes excluded from analysis of microsyntenic conservation in UR(B) regions apparently do not have annotated homologs in distant drosophilids, and the proportion of excluded genes is higher in UR(B).

### Genes without Human Homologs Evolve Faster in UR(B) Compared with the Adjacent Regions

**Fig. 1.—:**
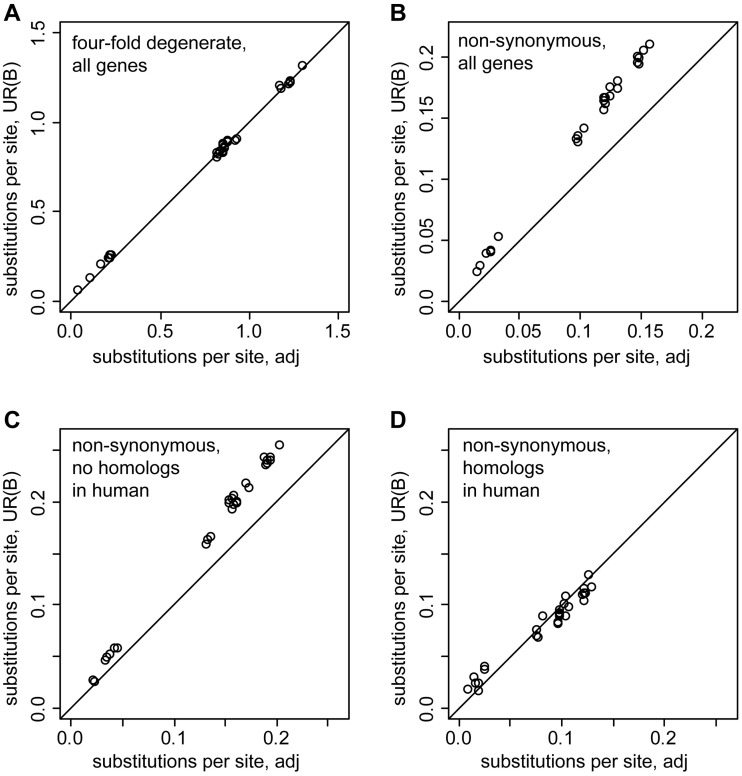
Comparison of phylogenetic distances for genes located in UR(B) and adjacent regions. The ORF alignments for all genes in the indicated groups were merged into a single contig, and the pairwise distances (in substitutions per site) were calculated with MEGA4 using the Pamilo–Bianchi–Li method for all combinations of dm3, droSim1, droYak2, droEre2, droAna3, dp4, droPer1, droWil1 (see Materials and Methods for details). The pairwise distances for UR(B) genes are shown on the ordinate, the distance for the genes in the adjacent regions is on the abscissa. Black lines indicate the expected identical divergence in UR(B) and the adjacent regions. (*A*) Divergence at 4-fold degenerate sites is essentially identical in UR(B) and the adjacent regions. (*B*) Genes in UR(B) display more nonsynonymous substitutions than the adjacent genes. (*C*) Nonsynonymous sites of genes without human homologs show a higher divergence in UR(B) than in the adjacent regions. (*D*) The divergence at the nonsynonymous sites of the genes with human homologs is very similar in UR(B) and the adjacent regions. Note that the protein sequences of the genes with human homologs evolve slower than the genes without human homologs shown on the panel (*C*).

We compared the substitution rate of genes in UR(B) and adjacent regions using the whole-genome alignment available on the UCSC Genome Browser site. For every UR(B) region we selected the same number of genes in adjacent regions, half upstream and half downstream, excluding genes overlapping with other UR(B). In total, we used 891 “adjacent” genes (supplementary data set S2, Supplementary Material online). Only species from the *Sophophora* subgenus were used to minimize the effect of nonconserved genes that are absent in the distant drosophilids. For example, 97% of FlyBase Genes (version 5.12) on chromosomes X, 2, and 3 are aligned to the *D. virilis* genome compared with 93% for genes in UR(B). We selected one transcript isoform per gene (first in alphabetical order) assigned to UR(B) and adjacent regions, extracted the ORFs alignments, and estimated the pairwise distances for concatenated alignments using MEGA4 (Pamilo and Bianchi 1993; Tamura et al. 2007) for several groups of genes (fig. 1).

The pairwise phylogenetic distances calculated for 4-fold degenerate sites are essentially identical between genes in UR(B) and adjacent regions (fig. 1*A*), whereas the distances estimated for nonsynonymous substitutions are on average 1.4 times greater in UR(B) (fig. 1*B*). Genes without human homologs evolve on average 1.3 times faster in UR(B) (fig. 1*C*), whereas the substitution rate at nonsynonymous sites of genes with human homologs is essentially identical in UR(B) and the adjacent regions (fig. 1*D*). Our results show that the genes without homologs in distant species evolve faster in UR(B) compared with the adjacent regions.

### Genes without Homologs in Distant Species Encode Shorter Proteins

An average length of proteins encoded by 933 UR(B) genes is 477 aa (amino acid residues) compared with 510.5 aa for 891 genes in the adjacent regions. Gene *dp* (*dumpy*, *CG33196-RA*) located in UR(B) 25A1-4 encodes an extremely long (22,971 aa) protein. Without Dumpy, the average length of proteins produced by UR(B) genes is 452.9 aa. The median length of proteins in UR(B) is 334 aa compared with 410 aa for the adjacent regions.

**Fig. 2.—:**
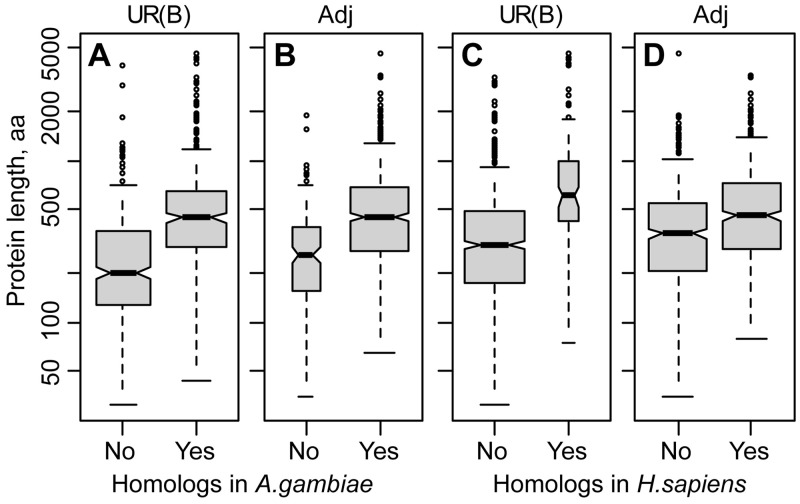
Comparison of protein length of genes with and without homologs in distant species. (*A*) *D. melanogaster* genes located in UR(B) with (Yes) or without (No) homologs in the mosquito genome. (*B*) Genes in the adjacent (Adj) regions with (Yes) or without (No) homologs in the mosquito genome. (*C*, *D*) The genes in UR(B) and the adjacent regions were split according to conservation in the human genome. The box width is drawn proportional to the square root of the gene number on each panel. The 22,971-aa-long protein encoded by *dp* (*dumpy*) and located in UR(B) was excluded from the data set. The shaded boxes show the first and the third quartiles. Solid lines in the middle of the gray boxes correspond to the median. The whiskers extend to the most extreme data point which is no more than 1.5 times the interquartile range from the box (*R* default). The nonoverlapping notches indicate strong evidence for a statistical difference between the medians.

We checked whether the length of the proteins correlates with the conservation status in distant species. The genes without homologs in mosquito or human encode shorter proteins compared with the genes with homologs (fig. 2). For example, the average protein length of the 829 UR(B) genes without human homologs is 397 aa compared with 1,114 aa for the 104 proteins conserved in human. After exclusion of the 22,971-aa-long Dumpy protein, the average length of UR(B) proteins with human homologs declines to 902 aa, but is still 2.3 times greater than the average length of proteins without human homologs (Wilcoxon test, *P* = 2.2E-16). Nonoverlapping notches on box-and-whiskers plots of proteins with and without homologs in the distant species (fig. 2) also suggest that the difference in protein length between the two groups is statistically significant.

The genes with homologs in distant species located in UR(B) encode longer proteins compared with the adjacent regions. For example, the average length of the proteins with human homologs is 902 aa in UR(B) versus 595 aa in the adjacent regions (1.5-fold difference, Wilcoxon test, *P* = 0.0005). The opposite is true for the genes without homologs in distant species. On average, proteins without human homologs are slightly shorter in UR(B) than in the adjacent regions, 397 and 427 aa, respectively (1.1-fold difference, Wilcoxon test, *P* = 0.0009).

Our results indicate that the UR(B) regions are enriched with fast-evolving genes encoding short proteins. The genes with homologs in distant species have longer proteins in UR(B) compared with the adjacent regions, whereas the nonconserved genes in UR(B) tend to encode shorter proteins.

### UR(B) Are Enriched with Highly Conserved Noncoding Sequences

The UR(B) regions are enriched with fast-evolving genes but at the same time in many regions the gene order is conserved in drosophilids (Andreyenkova et al. 2013). It is possible that some genes in UR(B) are controlled by distant regulatory elements (Montavon et al. 2011) and are very sensitive to chromosomal rearrangements. Some of the known drosophila genes with distant regulatory elements such as homologs of Hox genes (*Ubx*, *abd-A*, and *Abd-B*) are mapped to UR(B). Such a network, if it exists, might be associated with highly conserved nonexonic sequences. For example, in mammals the UCEs (Bejerano et al. 2004) are located in gene deserts resistant to chromosomal rearrangements (Ovcharenko et al. 2005) and many UCEs act as distant enhancers (Pennacchio et al. 2006). We analyzed the distribution of different conserved nonexonic sequences in UR(B).

**Fig. 3.—:**
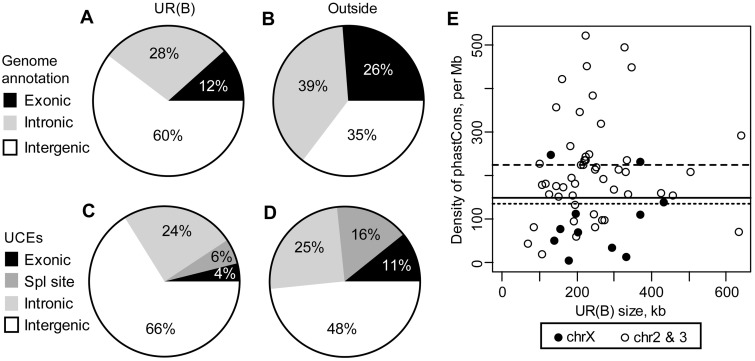
Distribution of conserved sequences in UR(B). (*A*) Genomic annotation based on FlyBase Genes 5.12 for the UR(B) and (*B*) the remaining part of chromosomes X, 2, and 3. (*C*) Distribution of Sophophora UCEs in UR(B) and (*D*) the remaining part of the genome. “Exonic” UCEs do not include UCEs overlapping splice sites. (*E*) Density of nonexonic phastCons elements with score greater than 600 in UR(B). The solid black line shows the average density of the phastCons600 elements in the genome, dashed and dotted lines indicate the average density in UR(B) and the remaining part of the genome, respectively.

Sophophora UCEs were identified as sequences identical over at least 100 nucleotides in several species from the *Sophophora* subgenus (Makunin et al. 2013). Approximately 19% of the Sophophora UCEs (414 out of 2,124) are located in UR(B), 1.6 times more than expected from a random distribution in the genome (Chi-squared test, *P* = 1E-22). UCEs’ density is very high outside of genes, and intergenic regions occupy a higher proportion of UR(B) compared with the rest of the genome (fig. 3), making these regions the biggest contributors of UCEs and partially explains the excess of UCEs in UR(B) but the largest statistically significant difference is observed in the intronic regions (table 2). This is somewhat surprising considering that the proportion of intronic regions is smaller in UR(B) (fig. 3), and that the intronic UCEs have a modest preference for genes with homologs in distant species (data not shown) whereas the proportion of such genes in UR(B) is significantly smaller.

**Table 2:** Enrichment of Conserved Nonexonic Sequences in UR(B)

	UCEs		phastCons600		HCNEs	
	Introns	Intergenic	Introns	Intergenic	Introns	Intergenic
Observed	101	274	797	2,137	500	1,435
Expected^a^	49.6	215.9	414.3	1,735.9	228.3	1,007.5
Exp./Obs.	2.0	1.3	1.9	1.2	2.2	1.4
Chi-squared test, *P* value	1.9E-14	1.0E-05	7.4E-87	6.9E-27	1.2E-79	5.3E-51
Density, per Mb^b^	11.9	24.1	99.7	194.1	54.9	112.7

^a^Expected = density × length of the corresponding regions in UR(B).

^b^Density in all introns or intergenic intervals of the genome.

Out of 289 loci with the intronic UCEs in the drosophila genome, 46 (16%) are located in UR(B), whereas only 7% of all FlyBase genes are in UR(B) (Chi-squared test, *P* = 1.2E-9). Not only the proportion of genes with the intronic UCEs is higher in UR(B) but also the number of UCEs per gene is higher (2.2 intronic UCEs per gene in UR(B) vs. 1.8 in other regions).

Many intronic UCEs are located in long genes. The average length of 289 FlyBase genes with the intronic UCEs is 43 kb compared with 4.9 kb for the remaining genes. The average length of 46 genes with the intronic UCEs is even higher in UR(B), 51.6 kb. The significant overrepresentation of the intronic UCEs in UR(B) might be partially explained by a relative abundance of very long genes in these regions: UR(B) regions contain 7% of all genes (933 out of 13,570) but harbor 12% (39 out of 324) of genes over 40 kb in length (approximately average length of loci with the intronic UCEs).

We examined the distribution of the most conserved phastCons elements (Siepel et al. 2005). The transformed log-odds conservation strength of these sequences is recorded as a score in the corresponding track on the UCSC Genome Browser.

Out of 13,274 nonexonic phastCons elements with a score higher than 600 (phastCons600) 8,860 (67%) are intergenic, and 4,414 (33%) are intronic, whereas these regions occupy nearly equal fraction of the drosophila genome: 38% and 37%, respectively. In UR(B), both intronic and intergenic regions have more phastCons600 elements than expected (table 2). The highest enrichment is observed in introns, which is consistent with the enrichment of the Sophophora UCEs described above. The HCNEs (Engstrom et al. 2007) have a similar distribution (table 3).

**Table 3:** Short Noncoding RNAs in UR(B)

	Exonic	Intronic	Intergenic
miRNAs: Observed	1	2	8
Expected	1.4	10.9	18.8
Exp./Obs.	1.4	5.4	2.4
snoRNAs: Observed	0	3	0
Expected	0.8	16.8	11.0
Exp./Obs.	NA	5.6	NA
tRNAs: Observed	0	2	35
Expected	0.1	10.8	35.3
Exp./Obs.	NA	5.4	1.0

Note.—NA, not applicable.

As Sophophora UCEs, intronic phastCons600 elements have a very strong preference for long genes. For example, the average length of 111 genes with such sequences located in UR(B) is 37,244 bp compared with just 2,394 bp for the remaining 822 genes without intronic phastCons600 sequences.

Out of 1,058 genes with intronic phastCons600 sequences located on main chromosomes, 574 (54%) are conserved in the human genome compared with 43% of genes without such elements. In UR(B) out of 111 genes with intronic phastCons600 elements located in UR(B), 34 (31%) have human homologs compared with just 9% for genes without such sequences. The presence of the highly conserved intronic sequences correlates with conservation of genes in evolution.

The density of nonexonic phastCons600 elements varies nearly 100-fold (nearly 2 orders of magnitude difference) among UR(B) regions (fig. 3*E*), with 40 out of 60 UR(B) regions showing the density higher than the genomic average. In particular, ten UR(B) (supplementary data set S1, Supplementary Material online) have very high density, including UR(B) 89E1-4 (home for *Ubx*, *abd-A*, and *Abd-B* genes). Out of the ten UR(B) with lowest density of nonexonic phastCons600 elements, six are mapped to the X chromosome. The density of nonexonic phastCons600 elements in UR(B) regions on chromosome X is half that on the autosomes (104 and 220 elements per Mb, respectively). The results show that the majority of UR(B) have a high density of highly conserved nonexonic sequences. However, it is not an obligatory feature of all UR(B), especially those located on chromosome X (supplementary data set S1, Supplementary Material online).

### Noncoding RNAs in UR(B)

In mammals, some long noncoding RNAs span hundreds of kilobases and participate in various regulatory processes (Mattick and Makunin 2006). In drosophila, long noncoding RNAs (ncRNAs) such as *bxd* are known for several decades (Lipshitz et al. 1987). This particular ncRNA is mapped to UR(B) 89E1-4. We analyzed distribution of several known collections of ncRNAs in UR(B).

Annotated noncoding genes (FlyBase noncoding) are underrepresented in UR(B). Out of the 930 noncoding transcripts annotated on main chromosomes, only 61 (7%) overlap with UR(B). However, UR(B) regions harbor 15% of FlyBase noncoding bases indicating that long RNAs might be biased toward UR(B). The FlyBases noncoding genes annotation includes many short RNAs (snoRNA, small nuclear RNA, and microRNAs [miRNAs]) as well as numerous 5S rRNAs and some long ncRNA such as *bxd*. Some of these RNAs are clustered in the genome, for example, 5S rRNAs. We investigated the distribution of the most common types of ncRNAs.

Annotated miRNAs and snoRNAs are severely underrepresented in UR(B) regions (table 3). In addition, all 29 snoRNA candidates identified from 50 million reads on chromosomes X, 2, and 3 (Jung et al. 2010) are located outside UR(B). The tRNA genes are underrepresented in the UR(B) introns (table 3). The depletion of short RNAs in UR(B) is not limited to known classes of RNAs. For example, out of 44 class 3 candidate ncRNA (40 nt or longer) located on chromosomes X, 2, and 3 (Jung et al. 2010) none is mapped to UR(B). However, out of 35 putative short (<40 nt) ncRNAs identified in the same work, 6 (17%) overlap UR(B) including two candidates with very high read counts.

We examined the distribution of transcripts from recently a published collection of long intergenic noncoding RNA (lincRNAs) (Young et al. 2012). Out of 1,106 lincRNAs, 220 (20%) overlap with UR(B) for at least 50% of their length, nearly identical to the expected value based on proportion of intergenic sequences. The intergenic regions in UR(B) comprise 20% of this fraction in the drosophila genome but contain 25% of lincRNA bases. Still, lincRNAs cover only 3.3% of UR(B) bases.

Although lincRNAs are not enriched in UR(B), these regions show a modest preference for long noncoding RNAs. On average, lincRNAs occupy 2.2 kb in UR(B) compared with 1.7 kb in the remaining part of the genome (Wilcoxon rank sum test, *P* = 0.048). Still, only 5.5% of intergenic bases in UR(B) are covered by these long ncRNAs. Although lincRNAs might represent an important UR(B) component, their fairly small length in these regions does not support their involvement in maintenance of synteny between different drosophila species.

### Genes without Homologs in Distant Species Tend to Replicate Late in Cell Culture

**Fig. 4.—:**
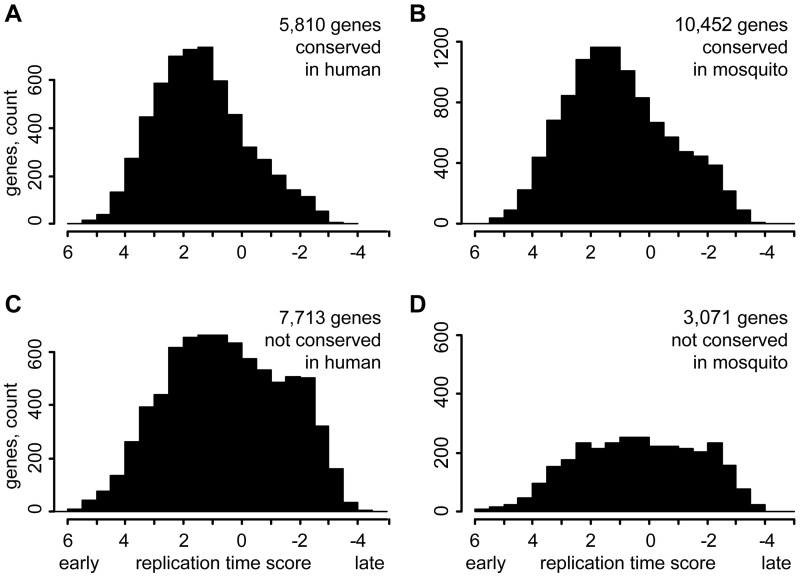
Replication time of genes with and without homologs in distant species. (*A*) Replication time in Kc cells of genes with similarity to human proteins. (*B*) Replication time of genes with homologs in the *Anopheles gambiae* genome. (*C*) and (*D*) Replication time of genes without homologs in human and mosquito, respectively. The replication time score was calculated as the average for all probes overlapping a gene. The genes without a replication score were not counted.

We checked whether gene conservation status in distant species correlates with the replication timing on the genome scale and compared the replication time of genes with and without homologs in distant species in two cell cultures, embryonic Kc and somatic Cl8 (Schwaiger et al. 2009). For every gene, we calculated an average replication score for all probes overlapping the corresponding locus. In embryonic Kc cells, the genes without homologs in the human or mosquito genomes are overrepresented in the late replicating fraction compared with the genes conserved in these species (fig. 4). A mean replication timing score of 5,810 genes conserved in the human genome is 1.4 compared with 0.5 for 7,713 nonconserved genes (*P* = 5.4E-146, Wilcoxon rank sum test). Exclusion of genes located in UR(B) reduces the bias but it is still significant (data not shown). The genes without homologs in distant species also tend to replicate later in Cl8 cells derived from wing discs albeit the trend is less pronounced (supplementary fig. S1, Supplementary Material online). The Cl8 cell culture was originated from males, and in drosophila males the X chromosome replicates early due to dosage compensation (Schwaiger et al. 2009), so we excluded genes located on X chromosome from analysis of Cl8 data.

## Discussion

The late replicating URs have distinct structure and evolution pattern. The UR(B) regions have low gene density (Belyakin et al. 2010). Although the gene order in these regions is maintained in drosophilids (Andreyenkova et al. 2013), few UR(B) genes have homologs in distant species such as mosquito and human. The miRNA and snoRNA genes are underrepresented in UR(B). In the same time, the UR(B) regions are enriched in highly conserved noncoding sequences (table 2).

Almost every UR(B) region contains at least one long gene (supplementary data set S1, Supplementary Material online). The exceptions are eight UR(B) regions where the longest genes are under 10 kb, and only in three of these (100A1-2, 89A8-9, and 70A1-2) are the longest genes under 5 kb. However, these three UR(B) regions overlap longer syntenic blocks, or orthologous landmarks (von Grotthuss et al. 2010) that contain long genes next to the UR(B) regions. In fact, UR(B) 89A8-9 overlaps 24.3-kb-long *pxb* gene, but the gene is not assigned to UR(B) because less than 50% of its bases are covered by the UR(B). We can conclude that nearly all UR(B) regions either contain at least one long gene or have a long gene in a conserved syntenic block overlapping or adjacent to the UR(B) region.

The structure of UR(B) regions resembles “genomic regulatory blocks” (GRBs), regions with conserved gene order enriched with highly conserved nonexonic sequences (Engstrom et al. 2007). GRBs contain genes with complex regulatory architecture as well as “bystander genes” unrelated to the regulatory network. Maintenance of such regulatory architecture associates with the preservation of gene order in evolution. The microsynteny blocks overlapping with peaks (clusters) of HCNEs are longer and harbor more genes and independent gene anchors compared with the remaining syntenic blocks in the genome (Sahagun and Ranz 2012). GRBs may contribute to the conservation of gene order in diverse lineages across over 600 Myr of evolution (Irimia et al. 2012). The clustering is also reported for peaks of HCNEs (Sahagun and Ranz 2012). One of the possible mechanisms underlying such clustering might be linked with the preferential association of conserved syntenic blocks with nuclear periphery through LAMIN binding (Ranz et al. 2012) or extended regions of silent chromatin (Andreyenkova et al. 2013).

The UR(B) regions harbor 20% of lincRNAs annotated in the *D. melanogaster* genome. Some of the long ncRNAs located in UR(B) such as *bxd* participate in gene regulation (Petruk et al. 2006) but a role for the majority of these transcripts is unknown. Induced expression of a transgene inside of the intercalary heterochromatin leads to changes in replication timing, polytenization level, and chromatin structure in a broad area around integration site (Koryakov et al. 2012) indicating that the transcription in the intercalary heterochromatin can have a profound effects on a local genomic environment. Transcription from *P-*element insertions is required for manifestation of dominant *Ultraabdominal* alleles, whereas blocking the transcription reverts the mutant phenotype (Bender and Fitzgerald 2002). Transcription through the silenced regulatory region results in its ectopic activation (Hogga and Karch 2002).

Late replicating regions packed with CNEs may serve as a “testing ground” for novel genes through expression in testis or transcriptional noise (Kaessmann 2010; Polev 2012). Such novel genes can be derived from lincRNAs or retroposed genes: Out of 97 potential functional retroposed genes identified in the drosophila genome (Bai et al. 2007), 18 are located in UR(B), 2.7 times more than expected (Chi-squared test, *P* = 5.5 E-06). Novel genes can be created through duplications, such as multiclusters “Osiris” and “Stellate” located in UR(B), and duplication hotspots are biased to late replicating regions (Cardoso-Moreira et al. 2011). Novel genes can be derived from transposable elements (Sinzelle et al. 2009), and mobile elements can contribute regulatory elements and promoters to genes. The latter phenomenon is fairly common in mammals (Peaston et al. 2007) but also occurs in flies (Makunin and Iurlova 2010). The annotated transposable elements cover 4.8% of UR(B) bases compared with 2.4% for neighboring regions, and the biggest difference observed for the long terminal repeat retrotransposons. We can describe the UR(B) regions as evolution hotspots with significant gene turnover. The URs represent a subset of all late replicating regions of the drosophila genome (Zhimulev et al. 2003), and we believe that our findings can be extrapolated for all late replicating domains.

## Supplementary Material

Supplementary figure S1 and data sets S1 and S2 are available at *Genome Biology and Evolution* online (http://www.gbe.oxfordjournals.org/).

Supplementary Data
