# Supplementary material for: Underreplicated Regions in Drosophila melanogaster Are Enriched with Fast-Evolving Genes and Highly Conserved Noncoding Sequences
Source: Genome Biol Evol. 2014 Jul 24;6(8):2050–60. doi: 10.1093/gbe/evu156 (PMC4159006; doi:10.1093/gbe/evu156)
Supplement: Supplementary Data [file supp_6_8_2050__index.html]

Under-replicated regions in D. melanogaster are enriched with fast evolving genes and highly conserved noncoding sequences — Underreplicated Regions in Drosophila melanogaster Are Enriched with Fast-Evolving Genes and Highly Conserved Noncoding Sequences — Supplementary Data 

# Underreplicated Regions in *Drosophila melanogaster* Are Enriched with Fast-Evolving Genes and Highly Conserved Noncoding Sequences

## Supplementary Data

files

**Files in this Data Supplement:**

- Supplementary Data - pdf file
- Supplementary Data - xls file
- Supplementary Data - xls file
