# Supplementary material for: Underreplicated Regions in Drosophila melanogaster Are Enriched with Fast-Evolving Genes and Highly Conserved Noncoding Sequences
Source: Genome Biol Evol. 2014 Jul 24;6(8):2050–60. doi: 10.1093/gbe/evu156 (PMC4159006; doi:10.1093/gbe/evu156)
Supplement: Supplementary Data [file supp_evu156_supplementary_figure_S1.pdf]

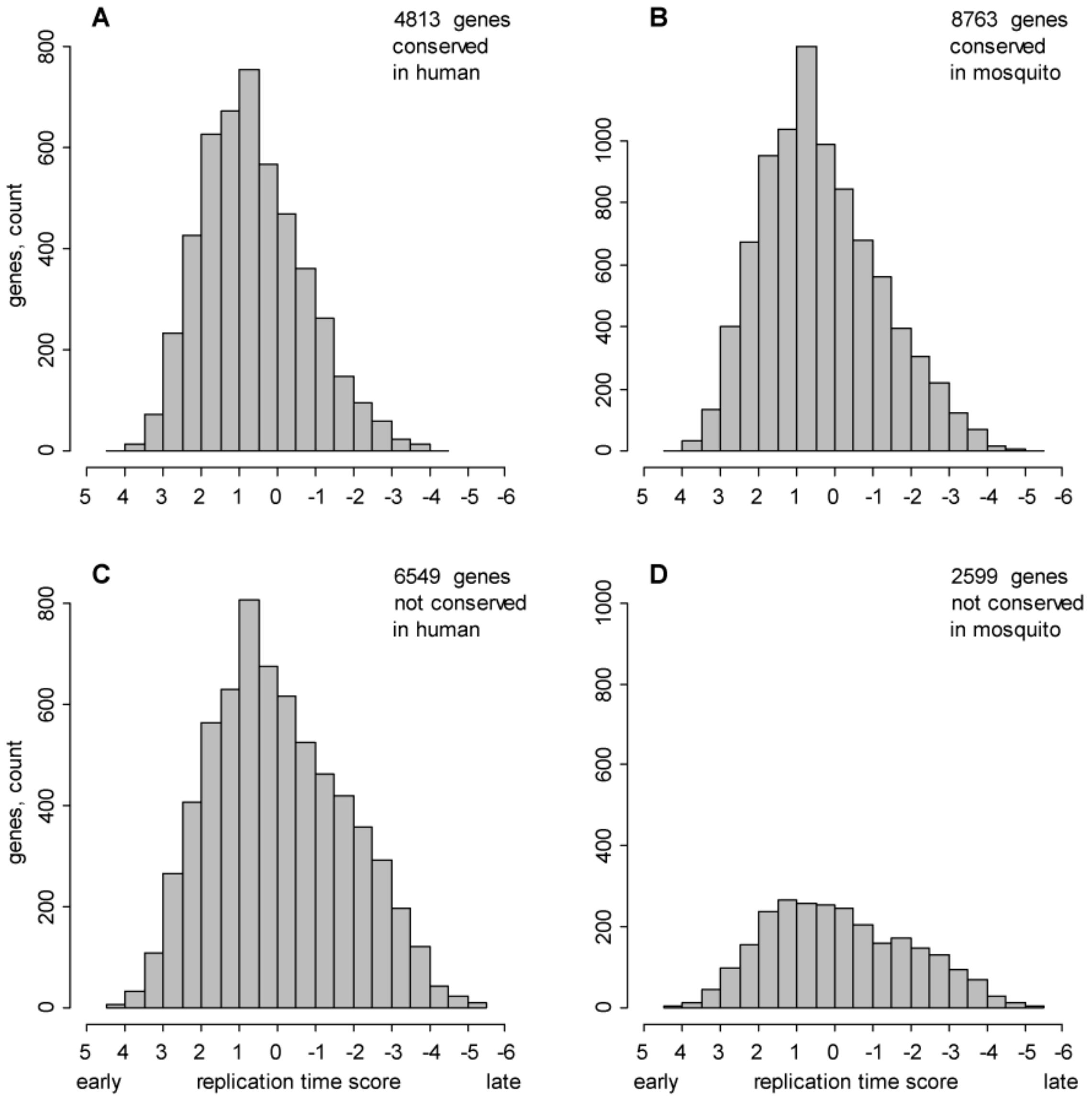

Supplementary fig. S1. Replication timing for genes on chromosomes 2 and 3 in Cl8 cells. (*A*) Genes with homologs in the human genome. (*B*) Genes with homolgs in the mosquito genome. (*C*) Genes without human homologs. (*D*) Genes without mosquito homologs.
